# Characterization of C-type lectins reveals an unexpectedly limited interaction between *Cryptococcus neoformans* spores and Dectin-1

**DOI:** 10.1371/journal.pone.0173866

**Published:** 2017-03-10

**Authors:** Naomi M. Walsh, Marcel Wuthrich, Huafeng Wang, Bruce Klein, Christina M. Hull

**Affiliations:** 1 Department of Biomolecular Chemistry, University of Wisconsin-Madison, School of Medicine and Public Health, Madison, Wisconsin, United States of America; 2 Department of Medical Microbiology and Immunology, University of Wisconsin-Madison, School of Medicine and Public Health, Madison, Wisconsin, United States of America; 3 Department of Pediatrics, University of Wisconsin-Madison, School of Medicine and Public Health, Madison, Wisconsin, United States of America; University of Michigan Health System, UNITED STATES

## Abstract

Phagocytosis by innate immune cells is an important process for protection against multiple pathologies and is particularly important for resistance to infection. However, phagocytosis has also been implicated in the progression of some diseases, including the dissemination of the human fungal pathogen, *Cryptococcus neoformans*. Previously, we identified Dectin-1 as a likely phagocytic receptor for *C*. *neoformans* spores through the use of soluble components in receptor-ligand blocking experiments. In this study, we used gain-of-function and loss-of-function assays with intact cells to evaluate the in vivo role of Dectin-1 and other C-type lectins in interactions with *C*. *neoformans* spores and discovered stark differences in outcome when compared with previous assays. First, we found that non-phagocytic cells expressing Dectin-1 were unable to bind spores and that highly sensitive reporter cells expressing Dectin-1 were not stimulated by spores. Second, we determined that some phagocytes from Dectin-1^-/-^ mice interacted with spores differently than wild type (WT) cells, but the effects varied among assays and were modest overall. Third, while we detected small but statistically significant reductions in phagocytosis by primary alveolar macrophages from Dectin-1^-/-^ mice compared to WT, we found no differences in survival between WT and Dectin-1^-/-^ mice challenged with spores. Further analyses to assess the roles of other C-type lectins and their adapters revealed very weak stimulation of Dectin-2 reporter cells by spores and modest differences in binding and phagocytosis by Dectin-2^-/-^ bone marrow-derived phagocytes. There were no discernable defects in binding or phagocytosis by phagocytes lacking Mannose Receptor, Mincle, Card-9, or FcRγ. Taken together, these results lead to the conclusion that Dectin-1 and other C-type lectins do not individually play a major roles in phagocytosis and innate defense by phagocytes against *C*. *neoformans* spores and highlight challenges in using soluble receptor/ligand blocking experiments to recapitulate biologically relevant interactions.

## Introduction

Historically, therapeutic approaches in treating human disease have directly targeted the causative agents of disease (e.g. anticancer drugs, antimicrobials, etc.). Recently, however, immunomodulatory biologics that instead target and alter the host immune response have shown promise [1]. In particular, targeting the host mechanisms of phagocytosis has shown promising results for treating multiple pathologies, including stroke, certain anemias, cancer, and AIDS [2–4]. Developing similar methods to treat fungal diseases is appealing because current antifungal drugs often lack efficacy, cause off-target toxicity in the host, or are not readily available worldwide [5].

Phagocytosis of microbes by host immune cells is mediated by receptors that bind to immunoreactive moieties on microbes (Reviewed in [6]). The most well characterized of these interactions among fungi is the binding between the C-type lectin receptor Dectin-1 and the predominantly fungal carbohydrate β-(1,3)-glucan. Dectin-1 was discovered to be the major receptor responsible for phagocytosis of the pathogenic yeast *Candida albicans*, being both necessary and sufficient for uptake [7]. The absence of Dectin-1 or blocking it through the addition of soluble ligand causes a profound loss of *C*. *albicans* phagocytosis [8].

Another pathogenic yeast, *Cryptococcus neoformans*, is the most common cause of fatal fungal disease and causes life-threatening meningoencephalitis in ~1 million people per year worldwide. Limited availability of effective antifungal drugs contributes to a mortality rate of ~600,000 patients annually [9]. Infection with *C*. *neoformans* occurs through inhalation of spores and/or yeast into the lungs from which it disseminates to the central nervous system (CNS) [10,11]. While the mechanism of dissemination is not well understood, *C*. *neoformans* is a facultative intracellular pathogen that can survive inside macrophages, and there is a positive correlation between phagocytosis of cryptococcal isolates and virulence [12,13]. Thus, a Trojan horse model by which *Cryptococcus* disseminates to the CNS inside immune cells has been proposed [14]. Given these relationships, understanding the mechanisms of phagocytosis is critical to determining the pathological progression of cryptococcal disease and ultimately developing new therapeutics (including immunomodulatory agents) to prevent disseminated cryptococcosis.

Dectin-1 was identified previously as a high likelihood receptor for interactions with *C*. *neoformans* spores via experiments in which the interactions between the receptor and spores were disrupted using three different methods: First, excess soluble ligand in the form of laminarin (mixed β-1,3- and β-1,6-glucan polymer) was introduced to saturate Dectin-1 binding sites on macrophages; second, the binding pocket of Dectin-1 on macrophages was blocked using a neutralizing antibody; third, the β-glucan ligand on the surface of spores was blocked through the addition of soluble Fc-Dectin-1 chimeric receptor protein.

Each of these approaches resulted in an approximately 50–65% reduction in phagocytosis [15]. These results, along with the observation that soluble Fc-Dectin-1 chimeric receptor protein was able to bind to spores as assayed by fluorescence microscopy, led to the conclusion that Dectin-1 was very likely required for normal levels of phagocytosis of *C*. *neoformans* spores by phagocytes. One caveat to drawing this conclusion, however, is that these kinds of blocking experiments have been employed frequently in testing host-pathogen interactions, and there are many instances of conflicting results. For example, Dectin-1 was reported to be an important receptor for *Aspergillus fumigatus* conidia phagocytosis, because blocking this interaction through the addition of excess ligand (laminarin) led to a significant reduction in uptake [16]. However, a subsequent study carried out under very similar conditions showed that blocking Dectin-1 with laminarin caused no significant change in phagocytosis [17]. The only apparent differences between the studies were the strains of *A*. *fumigatus* and fluorescent labeling schemes for assessment, illustrating how small variations in experimental design can lead to large differences in results and conclusions made from blocking experiments. Similarly, it was concluded that mannose receptor played a role in phagocytosis of *Mycobacteria* species from experiments that blocked the receptor on phagocytes in culture with soluble mannans [18]. It was later discovered, however, that macrophages harvested from mannose receptor knockout mice phagocytosed *Mycobacteria avium* just as efficiently as macrophages from WT mice [19]. These conflicting results call into question conclusions drawn from blocking experiments and underscore the importance of assessing receptor/ligand interactions using methods that more accurately replicate a normal biological context.

Because in vitro blocking experiments can be inconsistent indicators of biologically relevant interactions, and lack the biological context of membrane-embedded receptors and surface-bound ligands, we revisited the role of Dectin-1 in spore binding and phagocytosis in the context of intact cells. Using an array of approaches, we discovered that Dectin-1 is an unlikely major player in *C*. *neoformans* spore phagocytosis by phagocytes (macrophages and dendritic cells). Our findings call into question the use of in vitro blocking experiments to conclusively determine relevant interactions between microbes and specific receptors and suggest that accurate assessments of host-pathogen interactions require intact cells and a representative in vivo context.

## Methods

### Fungal strains and media

All strains were handled using standard techniques and media as previously described [20]. *C*. *neoformans* var. *neoformans* strain JEC20 (serotype D, mating type **a**), *C*. *neoformans* var. *neoformans* strain JEC21 (serotype D, mating type α), *C*. *neoformans* var. *neoformans* strain B3501 (serotype D, mating type α), *C*. *neoformans* var. *neoformans* strain B3502 (serotype D, mating type **a**), a GFP expressing *C*. *neoformans* var. *neoformans* strain B3501 (serotype D, mating type α) along with a backcrossed progeny (serotype D, mating type α), and *C*. *albicans* strain SC5314 were grown on yeast extract peptone dextrose (YPD) agar plates at 30°C and stored at 4°C. For all experiments with *C*. *neoformans* yeast, a 1:1 mixture of **a**:α mating type was used.

### *C*. *neoformans* spore isolation

Spores were isolated as described previously [21]. Briefly, yeast of both mating types were grown on YPD for 2 days, mixed in a 1:1 ratio in PBS and spotted on dried V8 plates. After 5 days, the crosses were suspended in 65% Percoll and 1X PBS, a gradient was formed by centrifugation for 20 minutes at 4000 RPM. Spores were recovered from the bottom of the gradient. Yeast contamination was assessed microscopically using a hemacytometer. Preparations of >99% and >95% purity for JEC20xJEC21 and B3501xB3502 spores, respectively, were used for experiments.

### CHO-K1 cell line constitutively expressing Dectin-1-HA

cDNA for the murine Clec7a (a.k.a Dectin-1) gene was purchased from Thermo Scientific Open Biosystems. Restriction sites for AgeI and BamHI were added to the 5’ and 3’ ends of the gene and the stop codon was removed using PCR. The resulting fragment was cleaved and ligated into the pSelect-CHA-blasti plasmid from Invivogen to generate a Dectin-1 protein with a C-terminal hemeagglutinin (HA) tag. The sequence-verified plasmid and an empty vector control were transfected into CHO-K1 cells (purchased from ATCC; cell line number CCL-61) with Lipofectamine 2000 (Life Technologies), and cultures were maintained in the presence of 10μg/mL blasticidin for five weeks. Stable transformants were stained with a primary mouse α-HA antibody (ThermoFisher Scientific, clone 2–2.2.14) and a secondary donkey α-mouse IgG conjugated to Cy3 (Jackson ImmunoResearch, code 715-165-150) and sorted to select for cells expressing Dectin-1 HA. Proper folding and function of the recombinant Dectin-1 was verified via recognition with a FITC-conjugated α-Dectin-1 antibody (Acris Antibodies, clone 2A11), and the ability of Dectin-1-HA cells to recognize and bind *Candida albicans*.

### CHO-K1 binding assays

CHO-K1 cells transfected with empty vector or the Dectin-1-HA plasmid were plated on glass slides, allowed to adhere, and co-cultured with calcofluor white-labeled fungal cells (heat killed or live) for 2 hours at a ratio of 100:1 (fungal cells to mammalian cells) at 37°C and 5% CO_2_. The slides were rinsed three times with cold PBS to remove unbound fungal cells, fixed with 4% paraformaldehyde overnight, and stained with a primary mouse α-HA antibody (ThermoFisher Scientific, clone 2–2.2.14) and a secondary donkey α-mouse IgG conjugated to Cy3 (Jackson ImmunoResearch, code 715-165-150). Cells were visualized using epifluorescence on a Zeiss Axioskop 2 Plus microscope at 1000X magnification. To quantify CHO cell association with fungal cells, a minimum of 300 CHO cells per condition was visualized and scored for the presence of associated fungi. A Fisher’s Exact Test was conducted to determine statistical significance [22].

### CLR reporter cell construction, stimulation and binding by fungi

B3Z and BWZ reporter cells were constructed previously as described [23,24]. Reporter cells were cultured in 96 well plates and stimulated for 18 hours with heat-killed fungal cells at 37°C and 5% CO_2_. Experiments conducted with *C*. *neoformans* were carried out at ratios varying from 1 to 300 (fungal cells to reporter cells), *C*. *albicans* stimulation was carried out at a ratio of 30:1 (yeast:reporter cells). Following stimulation, ß-galactosidase activity was measured in cell lysates using chlorophenol red-β-D-galactopyranoside (CPRG) (Roche) as a substrate. Optical density (OD) at 560 nm was measured using OD at 620 nm as a reference. Statistical analysis was carried out using a Student’s t-test on two biological replicates with two technical replicates each. To assess binding fungal cell binding, reporter cells were pre-stained with carboxyfluorescein succinimidyl ester (CFSE) and incubated with Uvitex 2B labeled *C*. *neoformans* spores. Fungal cell binding was quantified using flow cytometry on a BD LSRII and gated to assess CFSE^+^ events that were also Uvitex 2B^+^.

### Bone-marrow cell harvest

Bone marrow cells were obtained from the femurs of individual WT and receptor knock-out mice. Femurs were flushed with 5 mL cold PBS and passed through a 70 μm filter and red blood cells (RBCs) were lysed. Bone marrow derived mononuclear phagocytes (including macrophages and dendritic cells) were then differentiated using one of two GM-CSF-based methods. Recent studies have shown that GM-CSF derivation of bone marrow cells results in a heterogeneous population of phagocytes [25]. While we have not assessed the phagocyte composition in our samples, for the purposes of clarity within this manuscript, we have named these separately derived cell populations based on protocols historically used to enrich for macrophages or dendritic cells. To prepare macrophage-enriched bone marrow-derived phagocytes, cells were washed, plated with 2x10^6^ per petri dish in DMEM+10% FBS with 3 ng/mL recombinant GM-CSF and cultured at 37°C and 5% CO_2_. On day 3, the medium was refreshed, and on day 7 adherent cells were harvested with trypsin and allowed to adhere overnight for in vitro assays.

To prepare dendritic cell (DC)-enriched bone marrow derived phagocytes we derived cells as described previously [26]. Briefly, 2x10^6^ cells were cultured in 10mL of RPMI+10%FBS, supplemented with 1X beta-mercaptoethanol (Gibco) and 20ng/ml of murine recombinant GM-CSF (cRPMI) in non-tissue culture petri-dishes at 37°C and 5% CO_2_. On day 3, plates were supplemented with 10 mL cRPMI, and on days 6, 8, and 10, ten mL of culture were centrifuged, resuspended in fresh cRPMI and returned to the plate. Non-adherent cells were harvested on day 10 through centrifugation and resuspension in RPMI+10% FBS (without supplementation).

### Alveolar macrophage harvest

Alveolar macrophages (AMs) were obtained by bronchoalveolar lavage from individual WT and knock-out mice, using 15 mice per condition. Mice were euthanized using CO_2_, and their lungs were lavaged 10 times with 1mL warmed PBS supplemented with 0.2mM EDTA. Cells were recovered from lavage fluid through centrifugation, RBCs were lysed, and remaining cells were washed 2X with RPMI+10% FBS. Cells were counted with a hemacytomer, plated in 96 well plates with 1.2x10^5^ cells per well, and allowed to adhere overnight. The identity of the harvested alveolar macrophages as SiglecF+CD11c+CD64+Ly6G- was confirmed using flow cytometry (S1 Fig).

### Microscopy-based phagocytosis assays

Macrophage-enriched bone marrow-derived phagocytes were plated on glass slides and allowed to adhere overnight. Calcofluor white-labeled fungal cells (either live or heat-killed) were added at a ratio of 100:1 and cultured for 4 hours at 37°C and 5% CO_2_, at which point cells were washed 3 times with PBS, fixed with 4% paraformaldehyde overnight, mounted, and visualized. A minimum of 100 phagocytes for each condition was assessed for the presence or absence of fungi. The percentage of fungus-associated macrophages was calculated by dividing by the number of fungus-associated phagocytes by the total number of macrophages.

To differentiate between phagocytosed and bound *C*. *neoformans* spores, DC-enriched bone marrow-derived phagocytes were plated in 16-well Nunc chamber slides with 2.5x10^5^ cells/well. 5x10^5^ GFP expressing *C*. *neoformans* spores were introduced to each well and incubated for 4 hours. Non-bound cells were then washed away, and remaining cells were incubated for an additional 2 hours. To stain any bound, extracellular fungal cells that had not been internalized, wells were treated with 250 μM calcofluor white for 1 minute. Cells were washed 3X with PBS, fixed, and mounted with coverslips for microscopy.

### Colony forming unit (CFU)-based phagocytosis assay

Macrophage-enriched bone marrow-derived phagocytes were co-cultured with *C*. *neoformans* spores or yeast for 4 hours at an MOI of 10:1 at 37°C and 5% CO_2_. *C*. *neoformans* that had not adhered or been phagocytosed was then removed by washing 3 times with PBS. Phagocytes were lysed with 0.01% Triton x-100 (a concentration known not to affect the viability of *C*. *neoformans*) to release intracellular *C*. *neoformans*; the lysate was serially diluted and plated on YPD to determine the number of macrophage-associated *C*. *neoformans*. After 3 days of growth at 30°C, colony forming units were counted, and the percentage of phagocyte association was calculated as (# CFUs from lysate)/(CFUs introduced).

### Flow cytometry-based phagocytosis assays

Flow cytometry was used to evaluate binding vs. internalization of *C*. *neoformans* spores. AMs or DC-enriched bone marrow-derived phagocytes were plated at 1.2x10^5^ and 2.5x10^5^ cells per well, respectively, in 96 well plates. Alvelolar macrophages were allowed to adhere overnight, whereas DC-enriched phagocytes were allowed to adhere for 1 hour. 5x10^5^ GFP-expressing *C*. *neoformans* spores were introduced to each well, and incubated for 4 hours. Unbound cells were then washed away, and remaining bound cells were incubated for an additional 2 hours. Wells were then stained with 250μM calcofluor white for 1 minute and washed 3X with PBS. Cells were released from wells by incubation in Accutase (BioLegend, Fisher Scientific) for 10 minutes. AMs and DC-enriched phagocytes were stained with a 1:200 dilution of anti-mouse CD11c conjugated to Alexa700 (BD biosciences) and DC-enriched phagocytes were further treated with a 1:200 dilution of anti-mouse CD11b conjugated to APC-Cy7 (BD Biosciences) for 15 minutes on ice in FACS buffer (2mM EDTA and 0.5% BSA in PBS), washed three times, and fixed in 2% formaldehyde in PBS. Fungal cell phagocytosis and binding were quantified using flow cytometry on a BD LSRII with AMs gated as CD11c+ and DC-enriched bone marrow-derived phagocytes gated as CD11b+CD11c+. Phagocytosis was defined as the percentage of phagocytes that were GFP+CFW-, and fungal cell binding alone was defined as GFP+CFW+ phagocyte-associated events. Because macrophages in particular can autofluoresce in the green channel, phagocytes incubated without *C*. *neoformans* spores were included for straining and gating controls (S7A and S7B Fig).

### Murine virulence assays

Virulence was assessed by infecting groups of 16–20 week old C57BL/6 background mice via intranasal instillation with 2.5x10^5^ B3501xB3502 *C*. *neoformans* spores in a volume of 50 μl PBS as described previously [15]. Mice were age matched with a minimum of 6 mice per group. Infected mice were monitored daily for rapid weight loss or signs of distress (e.g. labored breathing, altered gait, ruffed fur) at which point they were euthanized by CO_2_ asphyxiation. To determine fungal burdens, organs were harvested, homogenized, serially diluted, and plated onto YPD agar plates to count CFUs. All experiments were carried out in accordance with the recommendations in the Guide for the Care and Use of Laboratory Animals of the National Institutes of Health under a University of Wisconsin-Madison Institutional Animal Care and Use Committee-approved protocol. UW-Madison is an AAALAC accredited institution.

## Results

### Dectin-1 is not sufficient for whole cell binding to *C*. *neoformans* spores

**Fig 1:**
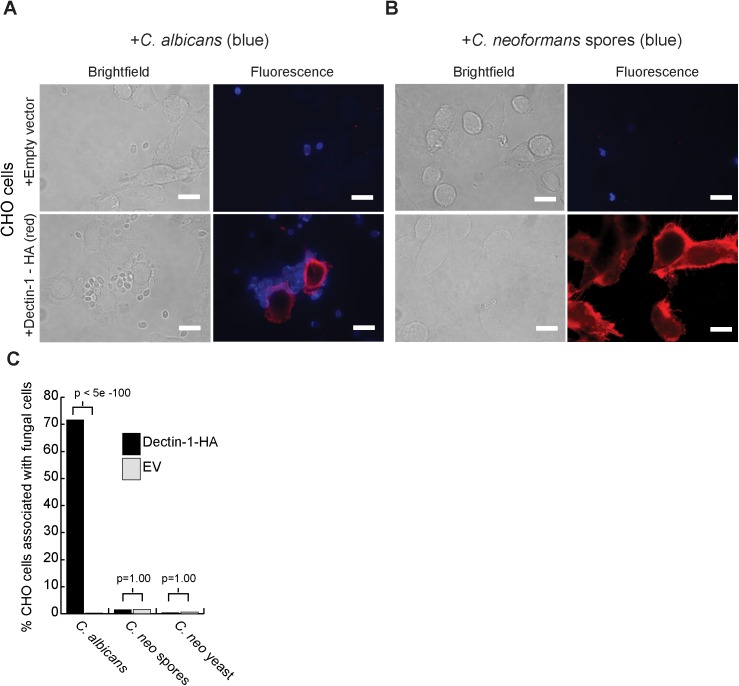
Dectin-1 alone is not sufficient to bind *C*. *neoformans* spores. CHO-K1 cells were engineered to express murine Dectin-1 (Clec-7a) with a C-terminal HA tag. Dectin-1-HA protein expression and localization were verified with an antibody directed against HA and conjugated to Cy3 (red). Visual assays were used to assess binding of heat-killed *C*. *albicans* yeast (**A**) and heat-killed *C*. *neoformans* spores (**B**). Fungal cells were stained with calcofluor white (blue). Cells were evaluated using both light and fluorescence microscopy at 1000X magnification. White bars represent 10 μm. **(C)** Interactions between CHO cells and live yeast or live spores were quantified in a representative biological replicate (1 of 3) by assessing a minimum of 300 CHO cells (7–10 frames) per condition, and the change in association was compared in the presence or absence of Dectin-1-HA for each fungal cell type using a Fisher’s Exact Test for statistical analysis (bracket values). X-axis shows fungal cell types. Y-axis represents % CHO cells associated with fungi. Black bars represent Dectin-1-HA expressing cells. Gray bars represent cells harboring an empty expression vector (EV).

We observed previously that a soluble Dectin-1-Fc chimeric protein bound to *C*. *neoformans* spores, suggesting that Dectin-1 is a biologically relevant receptor for spore interactions [15]. In blocking experiments, spores pre-treated with Fc-Dectin or alveolar macrophages pre-treated with an antibody against Dectin-1 prior to co-culture of spores and macrophages led to a 66% and 60% reduction in phagocytosis of spores, respectively [15]. However, given the often-conflicting results observed with blocking experiments in other fungal systems, we considered that Dectin-1 could be contributing to binding in vitro but be incapable of binding spores when embedded in a cell membrane due to topological interference. To determine whether or not spore binding occurs via Dectin-1 in the biological context of intact cells, we established a stable cell line of Chinese Hamster Ovary (CHO) cells expressing murine Dectin-1 with a c-terminal hemagglutinin (HA) tag. Dectin-1 expression and proper folding of the resulting protein was verified by the ability of an antibody directed against Dectin-1 (not the HA tag) to bind to the surface of cells transfected with the Dectin-1-HA construct (S2 Fig). Dectin-1-HA has also been used previously in other studies assessing Dectin-1 binding, indicating that the HA tagged protein would function properly [27,28]. Because Dectin-1 has been shown previously to be sufficient for binding *C*. *albicans*, we used *C*. *albicans* as a positive control for Dectin-1 binding. As expected, CHO cells expressing Dectin-1-HA readily bound *C*. *albicans* yeast (Fig 1A, bottom panels), and CHO cells transfected with an empty vector did not (Fig 1A, top panels). In contrast, there was no binding of *C*. *neoformans* spores (Fig 1B) or yeast (S3 Fig) to either of the CHO cell lines (empty vector or Dectin-1-expressing). Quantification of this interaction revealed that 70% of CHO cells expressing Dectin-1-HA bound *C*. *albicans*, and this association was lost in the absence of the receptor (p = 5x10^-100^). *C*. *neoformans* spores and yeast showed no binding to CHO cells and the data were statistically identical in the presence or absence of Dectin-1-HA (p = 1.0) (Fig 1C). There were no differences in binding between live and heat-killed fungal cells in either the qualitative or quantitative association assays. From these data, we conclude that in this assay Dectin-1 alone is not sufficient to bind *C*. *neoformans*. This contrasts sharply with the ability of soluble Dectin-1 protein to bind to the surface of spores. Given these conflicting findings, we entertained three possibilities for the role of Dectin-1 in spore binding: 1) Dectin-1 could recognize and bind *C*. *neoformans* spores, but the affinity of the interaction is insufficient to maintain whole-cell (CHO cell) binding of spores; 2) Dectin-1 may bind spores, but in the context of cells it requires a binding partner (not present on CHO cells) to do so, or 3) Dectin-1 may *not* be involved in spore binding between intact host cell and microbe, and previous observations of soluble Dectin-1 protein binding to spores do not reflect biologically relevant interactions.

### The Dectin-1 receptor alone does not recognize *C*. *neoformans*

**Fig 2:**
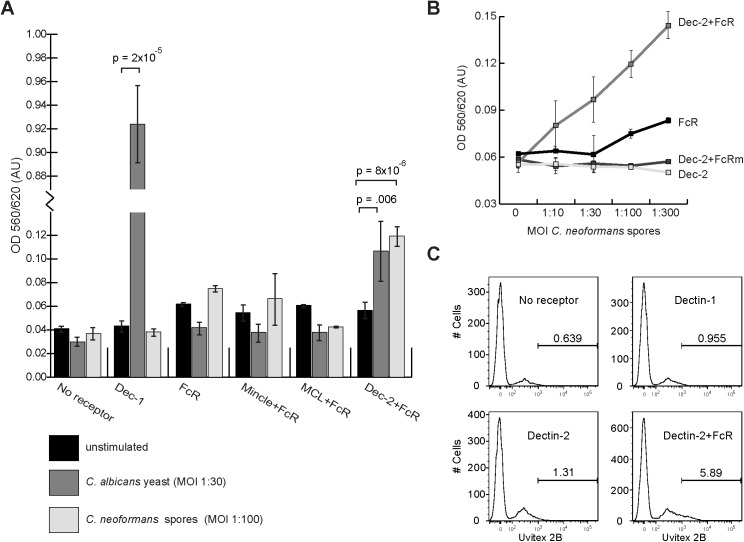
Dectin-1 alone does not recognize cryptococcal spores. (**A**) Reporter cells expressing mouse FcRγ chain only (FcR), Mincle and FcRγ chain together (Mincle+FcR), MCL and FcRγ chain together (MCL+FcR), Dectin-2 and FcRγ chain together (Dec-2+FcR), and Dectin-1-CD3ζ (Dec-1) were left unstimulated (black bars), stimulated with heat-killed *C*. *albicans* yeast (positive control, light gray bars), or stimulated with heat-killed *C*. *neoformans* spores (dark gray bars). After 18 hours of co-incubation, ß-galactosidase activity was measured using a colorimetric assay and expressed in absorbance units (AU) on the y-axis. Data shown are the mean ± the standard deviation of duplicate wells of a single experiment and are representative of two independent experiments. Statistical analysis was carried out using a Student’s t-test on two biological replicates that included two technical replicates each. **(B)** Weak activation of Dectin-2 increased with higher numbers of spores and depended on the presence of FcRγ (Dec-2+FcR). X-axis shows the ratio of fungal cells to reporter cells with *C*. *neoformans* spores. Y-axis shows absorbance units (AU). FcR = FcR chain only, Dec-2 = Dectin-2 alone, Dec-2+FcRm = Dectin-2 expressed with an inactive FcRγ mutant. **(C)** Quantitative association of *C*. *neoformans* spores with receptor-expressing cell lines. Histograms showing number of cells associated with Uvitex 2B-labeled spores in each receptor-expressing subline as indicated. X-axes represent a log scale of fluorescence; y-axes show cell number. Bars delineate range of fluorescence indicative of *C*. *neoformans* binding; numbers on bars are the percentage of the total number of cells associated with *C*. *neoformans* spores.

To test the possibility that the inability of Dectin-1-expressing cells to bind *C*. *neoformans* is due to a low-affinity interaction that exists but is not sufficient to sustain observable physical binding, we used a highly sensitive cell-based assay shown previously to identify receptor engagement [23,24]. This assay uses T hybridoma cells expressing an NFAT-lacZ reporter of ITAM signaling (BWZ cells), with individual reporter cell lines expressing different upstream receptors from mice (summarized in S4 Fig). Cultured reporter cells expressing Dectin-1 were incubated for 18 hours with fungal cells that had been heat-killed to avoid overgrowth of the reporter cell culture during the assay. Receptor signaling was evaluated by measuring β-galactosidase activity in cell lysates using chlorophenol red-β-D-galactopyranoside (CPRG) as a substrate. As expected, Dectin-1 reporter cells in the absence of fungal cells did not show any β-galactosidase activity over background and incubation with the known Dectin-1-interacting yeast *C*. *albicans* resulted in a 20-fold increase in β-galactosidase activity compared to unstimulated cells (p = 2.0 x 10^−5^ by Student’s t-test) (Fig 2A). In contrast, however, we observed no β-galactosidase activity over unstimulated Dectin-1 reporter cells in the presence of spores (Fig 2A). Similarly, no activity was detected upon incubation with *C*. *neoformans* yeast (S5 Fig). These data indicate that neither *C*. *neoformans* spores nor yeast engage the Dectin-1 receptor to stimulate the reporter, indicating that membrane-bound Dectin-1 alone on the cell surface is not capable of activation by *C*. *neoformans*.

Because Dectin-1 on its own did not appear to be bound or stimulated by *C*. *neoformans* spores, we hypothesized that a different receptor could be responsible for spore-phagocyte interactions. *C*. *neoformans* spores have been shown previously to harbor mannose on their cell surfaces [21]. We used the same receptor reporter system to assess two receptors known to recognize mannose derivatives, Dectin-2 and Mincle, and a third receptor, MCL, which has been show to recognize mycobacterial trehalose dimycolate (cord factor) [29]. We observed that in response to exposure to spores or yeast, no reporter activity was detected over background for either Mincle or MCL. In contrast, β-galactosidase activity reproducibly increased approximately 2-fold in reporter cells co-expressing Dectin-2 and its adapter FcRγ (p-value = 8x10^-6^) (but not in cells expressing either Dectin-2 or FcRγ alone) (Fig 2A and 2B). We concluded that *C*. *neoformans* spores bound Dectin-2/FcRγ and activated the reporter cell-signaling cascade.

Stimulation of Dectin-2 + FcRγ cells by *B*. *dermatitidis*, *H*. *capsulatum*, and *C*. *posadasii* previously resulted in signals approximately 5 times higher than our observations with *C*. *neoformans* spores and *C*. *albicans* yeast, indicating that Dectin-2 engagement can result in robust activation by other fungi [23]; our data indicate relatively weak receptor activation by *C*. *albicans* and *C*. *neoformans*. Weak interactions between Dectin-2 and *C*. *albicans* yeast have been reported previously [30,31] and were recapitulated in our assay (p-value = 0.006) (Fig 2A). The level of activation by *C*. *albicans* yeast and *C*. *neoformans* spores was similar. Numerous studies have evaluated the weak interaction between *C*. *albicans* yeast and Dectin-2, and although they have shown that Dectin-2 engagement by *C*. *albicans* stimulates cytokine expression, this interaction is not important for phagocytic uptake of *C*. *albicans* cells [31]. Previous studies with *C*. *neoformans* yeast have also detected modest interactions with Dectin-2 [31]. It is possible that we detected a weak interaction with a cryptococcal ligand that is mostly unexposed or internal to the spore surface [32]. As anticipated, reporter cells co-expressing an ITAM signaling-defective mutant of FcRγ chain (FcRm) and Dectin-2 failed to increase β-galactosidase activity even with increased numbers of fungal cells (Fig 2B).

In another measure of reporter cell binding, we incubated the reporter cells with spores labeled with the chitin-binding compound Uvitex 2B, washed away unbound spores, and assessed the population of cells using flow cytometry. Consistent with the β-galactosidase assay, a small percentage (5.89%) of reporter cells expressing Dectin-2 with FcRγ were positive for the Uvitex 2B signal, indicating an association with spores (Fig 2C). Neither Dectin-1 nor Mincle-expressing cells was associated with Uvitex 2B above background levels. Overall, these data lead to the conclusion that Dectin-1, MCL, and Mincle in the context of intact cells are unable to bind *C*. *neoformans* spores, whereas Dectin-2 exhibits weak, but reproducible interactions with spores.

### Absence of Dectin-1, Dectin-2, Mincle, or mannose receptor does not alter phagocyte association with cryptococcal spores

**Fig 3:**
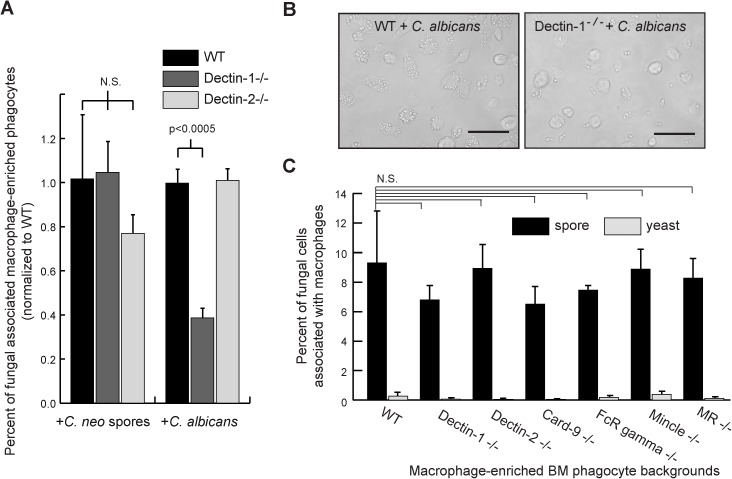
Absence of Dectin-1 or Dectin-2 does not alter association of cryptococcal spores with primary murine phagocytes. **(A)** Macrophage-enriched bone marrow-derived phagocytes from WT (black bars), Dectin-1 knockout (dark gray bars) and Dectin-2 knockout (light gray bars) mice were co-incubated with live *C*. *neoformans* spores or heat-killed *C*. *albicans* yeast, and microscopy was used to assess association of the fungal cells with the macrophages. X-axis shows the fungal cell types being assessed. Y-axis shows the percent of fungal-associated phagocytes relative to WT. Statistical analysis was carried out on 3 individual microscopic fields containing approximately 50 macrophages each. P-values are the result of a Student’s t-test. **(B)** Representative microscopy fields at 400X of heat-killed *C*. *albicans* co-incubated with WT and Dectin-1 knockout macrophage-enriched phagocytes (no difference was observed with *C*. *neoformans*). (Black bars = 50 μm) **(C)** Macrophage-enriched bone marrow-derived phagocytes from WT, Dectin-1^-/-^, Dectin-2^-/-^, Card-9^-/-^, FcRγ^-/-^, Mincle^-/-^ and MR^-/-^ mice were co-incubated with live *C*. *neoformans* spores (black bars) or live *C*. *neoformans* yeast (light gray bars) and association (binding + phagocytosis) frequencies determined by CFU analysis. X-axis shows the macrophage genotypes. Y-axis shows the percent of introduced fungal cells that were associated with the phagocytes. Data show the mean and standard deviations from a single experiment carried out in triplicate. An ANOVA with a Dunnett’s post-hoc test revealed that phagocyte association with spores was not statistically different from WT for any of the knock out phagocytes tested (p>0.05); results are representative of those obtained from 2–5 individual experiments for each knockout line compared to WT.

Having determined that no single receptor tested was sufficient for robust binding and receptor activation, we considered that these receptors were perhaps still necessary for interacting with *C*. *neoformans*, but required the participation of additional components present in their native cellular context. To test this possibility, we evaluated interactions between *C*. *neoformans* and primary murine phagocytes in culture harboring deletions in receptors of interest or their adapter proteins. Murine macrophage-enriched bone marrow-derived phagocytes from WT, Dectin-1 and Dectin-2 knockout mice were assayed for their ability to associate with spores. Live *C*. *neoformans* spores and heat-killed *C*. *albicans* (positive control) were incubated with the phagocytes for 4 hours, washed, and evaluated for associations using light microscopy. As expected, Dectin-1 knockout cells showed a significant (50%) decrease relative to WT in their association with *C*. *albicans*, and there were no differences between WT and Dectin-2 knockout cells with respect to *C*. *albicans* association (Fig 3A and 3B). For *C*. *neoformans* spores, we observed no significant differences in association with phagocytes in the absence of Dectin-1 or Dectin-2 (Fig 3A), indicating that Dectin-1 and Dectin-2 are not required for association with *C*. *neoformans* spores.

To determine potential roles for other receptors in spore interactions, we then tested an expanded set of receptor and adapter protein knockouts, including Mincle, mannose receptor, FcRgamma, and Card-9, and assessed association of *C*. *neoformans* spores and yeast with macrophage-enriched bone marrow-derived phagocytes. Phagocytes were incubated with live spores or yeast at an MOI of 10:1 for four hours, washed, lysed, plated onto YPD agar, and CFUs were used to determine what percent of the introduced fungal population had become associated with (either phagocytosed by or bound to) the macrophages. Neither of the receptor knockouts (Mincle or mannose receptor) showed any statistically significant change or pattern in association with spores as compared to WT macrophage-enriched phagocytes as assessed by an ANOVA with a Dunnett’s post-hoc test, indicating that these receptors are not necessary for spore association (Fig 3C) [33]. Furthermore, association was not altered in the absence of Card-9 or FcRγ, which serve as adapter proteins for a variety of C-type lectin receptors, including Dectin-1, Dectin-2, MCL, and Mincle, supporting our conclusion that these receptors are not essential for *C*. *neoformans* spore association. As has been observed previously, *C*. *neoformans* yeast did not readily associate with any of the macrophage-enriched phagocytes (Fig 3C). Overall, from these data we concluded that none of the receptors tested was necessary for overall macrophage-enriched bone marrow derived phagocyte association with *C*. *neoformans* spores.

### Dectin-1 and Dectin-2 play limited roles in internalization of *C*. *neoformans* spores by alveolar macrophages and dendritic cell-enriched bone marrow-derived phagocytes

Although overall *Cryptococcus*-phagocyte association was not altered by the absence of Dectin-1 or Dectin-2, the formal possibility remained that binding or internalization could be separately influenced by these receptors. To assess internalization and binding of spores by phagocytes we developed a flow cytometry-based phagocytosis assay. DC- enriched bone marrow-derived phagocytes or alveolar macrophages (AMs) harvested from WT, Dectin-1^-/-^ and Dectin-2^-/-^ mice were co-incubated with live spores expressing GFP. Unbound *C neoformans* were washed away, and the cultures were stained with calcofluor white to mark externally bound, but not phagocytosed fungal cells. Flow cytometry was used to quantitate different cell populations. AMs were identified through recognition by CD11c antibody, and DC-enriched phagocytes were identified by binding of both CD11c and CD11b antibodies. Phagocytes that were GFP+CFW- were defined as having completely internalized spores, whereas GFP+CFW+ phagocytes were classified as having at least one fungal cell bound but not phagocytosed. We verified our staining technique using microscopy and found that it effectively differentiated between internalized and bound *C*. *neoformans* spores for DC-enriched phagocytes harvested from all three genotypes (S6 Fig). Phagocytes incubated with no *C*. *neoformans* cells or with yeast (which resist binding and uptake) served as controls and resulted in a cell population that was both GFP and CFW negative, further validating our staining protocol (S7A and S7B Fig).

**Fig 4:**
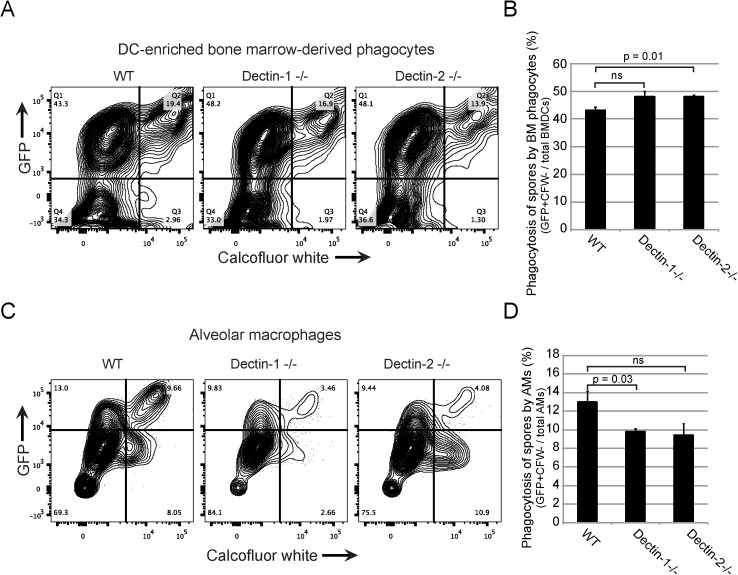
Dectin-1^-/-^ and Dectin-2^-/-^ phagocytes show modest changes in internalization of *C*. *neoformans* spores. Flow cytometry was used to differentiate and assess internalization and binding of *C*. *neoformans* spores by DC-enriched bone marrow-derived phagocytes and AMs. **(A)** Results of flow cytometry analysis using DC-enriched phagocytes shown as FACS plots and **(B)** bar graphs of spore internalization. DC-enriched phagocytes from WT, Dectin-1^-/-^, and Dectin-2^-/-^ mice show modest differences in internalization (GFP+CFW-) and binding (GFP+CFW+) of *C*. *neoformans* spores. ns = not significant (p = 0.1 by Student's t-test). **(C)** Results of flow cytometry analysis using AMs shown as FACS plots and **(D)** bar graphs of spore internalization. AMs from Dectin-1^-/-^ and Dectin-2^-/-^ mice show decreases in the phagocytosis of spores as compared to WT. ns = not significant (p = 0.09 by Student’s t-test). In all cases FACS plots were generated from experiments carried out in triplicate, with the exception of Dectin-2^-/-^ AMs, which were in duplicate. X-axes represent increasing calcofluor white signal. Y-axes represent increasing GFP signal. Bar graphs show average values, and error bars represent the standard error of the mean. X-axes indicate phagocyte background, and y-axes represent percent of phagocytes harboring spores.

When we assessed internalization events (GFP+CFW-) in the case of DC-enriched phagocytes, there was an apparent overall increase in phagocytosis of *C*. *neoformans* by both mutants relative to WT; however, the effect was small (43.3% for WT, 48.2% for Dectin-1^-/-^, and 48.2% for Dectin-2^-/-^) and Student's t-tests produced p = 0.01 for Dectin-2^-/-^ and p = 0.08 for Dectin-1^-/-^ (Fig 4A and 4B). We then assessed whether the small changes in overall internalization by Dectin-1 and Dectin-2 knockout DC-enriched phagocytes were result of altered spore binding or phagocytosis of spores that had been bound. Because all internalized spores first had to bind to phagocytes, percent binding included both internally and externally associated spores and was defined as (GFP+ phagocyte events/total phagocytes) x 100%. We found that WT, Dectin-1^-/-^, and Dectin-2^-/-^ were statistically identical in their ability to bind spores (62.7%, 65.1%, and 62%, respectively, p > 0.35) (S8A Fig). We also analyzed the role of Dectin-1 and Dectin-2 in phagocytosis of previously bound spores. For this purpose, phagocytosis was defined as DC-enriched phagocytes with internal *C*. *neoformans* spores divided by the total number of phagocytes that were able to bind spores (GFP+CFW-/GFP+). We found that Dectin-1^-/-^ and Dectin-2^-/-^ DC-enriched phagocytes were able to phagocytose bound spores slightly more efficiently (74.0% and 77.6%) than WT cells (69.1%) (S8B Fig). The observed increases in phagocytosis were statistically significant for both Dectin-1^-/-^ (p = 0.02) and Dectin-2^-/-^ (p = 0.003). There was no statistical difference among any of the phagocytes in binding or phagocytosis of *C*. *neoformans* yeast (data not shown). We conclude from these data that in DC-enriched bone marrow-derived phagocytess Dectin-1 and Dectin-2 do not play a significant role in spore binding, but may play a minor role in phagocytosis of bound spores, resulting in small increases in the number of internalized *C*. *neoformans* spores.

A similar experiment was carried out using AMs. The percentages of AMs harboring phagocytosed (internalized) spores were 13.0% for WT, 9.83% for Dectin-1^-/-^, and 9.44% for Dectin-2^-/-^ cells. For Dectin-1^-/-^ cells, the difference in a Student's t-test yielded a p-value of p = 0.03 (Fig 4C and 4D). Due to limited cell numbers, Dectin-2^-/-^ cells could be assessed only in technical duplicates, which may have contributed to greater error, resulting in a less significant p-value (p = 0.09). Once again, we analyzed the data to determine if the small decreases in phagocytosis were due to changes in the ability of the knockout AMs to bind, or to phagocytose *C*. *neoformans* spores. 22.7% of WT AMs were able to bind spores, whereas Dectin-1^-/-^ and Dectin-2^-/-^ AMs bound spores with a lower efficiency (13.3% and 13.5%, respectively) (S8C Fig). Similar to what was observed with DC-enriched phagocytes, we found that knockout AMs showed an increased ability to phagocytose bound spores with WT, Dectin-1^-/-^, and Dectin-2^-/-^ AMs exhibiting 57.3%, 73.9%, and 69.9% phagocytosis, respectively (S8D Fig) This increase in internalization was not sufficient to overcome the decreased ability to bind spores, and we conclude that the combination of these interactions resulted in an overall modest decrease in phagocytosis of *C*. *neoformans* by Dectin-1^-/-^ and Dectin-2^-/-^ AMs.

### Mouse survival is not significantly altered in Dectin-1^-/-^ or Dectin-2^-/-^ mice

**Fig 5:**
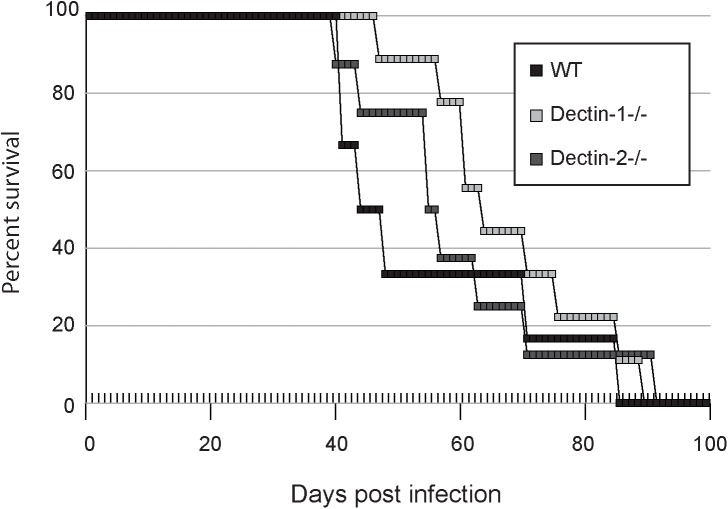
Mice lacking Dectin-1 or Dectin-2 do not show increased susceptibility or resistance to *C*. *neoformans*. Groups of WT (black), Dectin-1^-/-^(light gray), and Dectin-2^-/-^ (dark gray) mice were infected intranasally with 2.5x10^5^ *C*. *neoformans* spores and sacrificed when they became moribund. X-axis shows the number of days post-infection. Survival was tracked daily through the course of the infection. Y-axis shows the percentage of mice surviving in each group. There was no statistical difference between any of the mouse genotypes as assessed by a Log Rank (Mantel-Cox) test, (p > 0.1).

While it appeared that Dectin-1 and Dectin-2 play roles in binding and phagocytosis of spores in the context of DC-enriched bone marrow-derived phagocytes and alveolar macrophages, the differences were modest and did not approach the nearly 70% reduction in the uptake of spores by alveolar macrophages seen previously using blocking experiments for Dectin-1 [15]. Given this inconsistency, we considered that modest phenotypes could lead to large effects during active infections. To test whether Dectin-1 or Dectin-2 play any role in the ability of mice to survive an infectious challenge of *C*. *neoformans* spores, we introduced 2.5 x 10^5^ spores into the lungs of WT, Dectin-1^-/-,^ and Dectin-2^-/-^ mice via intranasal inoculation and monitored disease progression. We discovered that there was no statistical difference (Log Rank (Mantel-Cox) test, p > 0.1) in time to end point for mice lacking Dectin-1 or Dectin-2 compared to WT mice (Fig 5). Similarly, fungal burden in the lungs and brain at the endpoint of the experiment were not statistically different (data not shown), indicating that Dectin-1 and Dectin-2 do not play significant roles in the ability of *C*. *neoformans* to disseminate from the lung in spore-mediated infections. This agrees with previous studies showing that the absence of Dectin-1 or Dectin-2 neither significantly altered survival nor the lung fungal burden in mice challenged with *C*. *neoformans* yeast [32,34].

## Discussion

The data presented here show that in contrast to previous experiments using soluble components to block Dectin-1 and its ligand(s), cell-based assays did not indicate a substantial role for Dectin-1 in *C*. *neoformans* spore-mediated disease. It appears from our data that Dectin-1 contributes positively to spore binding and negatively to spore phagocytosis by alveolar macrophages, but these effects result in only modest increases in overall internalization. This effect on overall internalization is not seen with other phagocytes, nor does the absence of Dectin-1 lead to differences in disease outcome. These findings are mirrored with Dectin-2, and Dectin-2 similarly showed no significant effects in mouse virulence experiments. Furthermore, MCL, Mincle and mannose receptor were not essential for association with of *C*. *neoformans* spores. These findings make it highly unlikely that any individual C-type lectin is important in spore- (or yeast-) mediated cryptococcal disease in mice.

While Dectin-2 exhibited recognition of cryptococcal spores by highly sensitive reporter cells, this interaction was weak and did not fall into the range of known biologically relevant interactions in other systems. Previous reports indicate that intact *C*. *neoformans* yeast (B3501) are not recognized by Dectin-2 reporter cells, but activation of the reporter via Dectin-2 occurs upon lysis of the yeast [32]. Weak recognition of spores by Dectin-2 in our assays could be the result of two possibilities: First, exposure of an internal ligand by heat killing prior to the assay could allow a small amount of binding and activation (spores and yeast used in the reporter assays must be heat-killed to prevent fungal overgrowth and reporter cell lysis); second, Dectin-2 weakly recognizes surface ligands on spores that are not present or not exposed on encapsulated yeast. This hypothesis is supported by the fact that we observed a small amount of binding between intact spores and Dectin-2-expressing reporter cells via flow cytometry and small changes in spore phagocytosis by Dectin-2^-/-^ DC-enriched bone marrow-derived phagocytes (Fig 2C and Fig 4) as well as by previous findings that a carbohydrate recognition domain of Dectin-2 is able to recognize intact, capsule deficient yeast [35]. Although this weak interaction may occur in vivo, the absence of a phenotype in susceptibility to *C*. *neoformans* spores in Dectin-2 knockout mice, leads us to conclude that although Dectin-2 may play a role in recognition of *C*. *neoformans*, it is not essential for spore phagocytosis.

**Fig 6:**
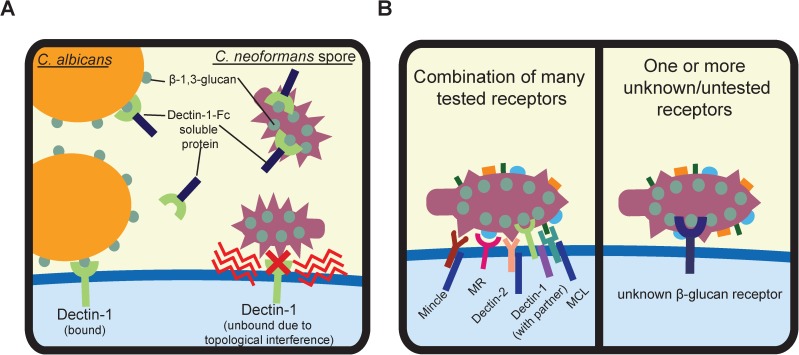
Model of receptor recognition of *C*. *neoformans* spores. **(A)** Soluble chimeric Dectin-1-Fc protein binds to β-glucan on *C*. *neoformans* spores, whereas Dectin-1 within the context of the cell membrane does not bind spores. This inability to bind could be due to topological interference between components on the spore coat and those on the host membrane and/or general inaccessibility of spore β-1,3-glucan to membrane-bound Dectin-1. In the case of *C*. *albicans*, β-1,3-glucan is exposed in such a way that both soluble and membrane-bound Dectin-1 can bind to it and engage downstream signaling. **(B)** Two overarching models of *C*. *neoformans* spore phagocytosis. Numerous Contributors (left): Although none of the receptors tested in these studies contributed significantly to spore phagocytosis, it is possible that many diverse receptors, including those tested, could each mediate weak spore interactions that facilitate phagocytosis. Unidentified Receptor(s) (right): It is possible that one or more currently unidentified or unknown receptors could be responsible for mediating the uptake of spores.

The observation that Dectin-1 did not interact with *C*. *neoformans* spores in the majority of our experiments was surprising given our previous findings that soluble chimeric Fc-Dectin-1 receptor bound spores and that blocking Dectin-1-spore interactions with excess ligand, soluble chimeric receptor, or Dectin-1 neutralizing antibody resulted in a striking reduction in phagocytosis [15]. We did observe a small but significant decrease in phagocytosis (from 13 to 10%) in primary alveolar macrophages, suggesting that Dectin-1 may play a role in phagocytosis in this cell type. However, this contribution to phagocytosis by Dectin-1 does not align with the 50–65% reduction in phagocytosis observed in previous blocking experiments. One explanation for this discrepancy is that the addition of excess ligand in the in vitro blocking experiments could have blocked other glucan receptors necessary for uptake of spores. Alternatively, the presentation of β-glucan on spores may be such that membrane-bound Dectin-1 is unable to robustly engage with it due to topological interference, whereas soluble chimeric receptor was able to bind and perhaps reduce phagocytosis mediated by other receptors via β-glucan on spores (Fig 6A). Finally, any of these reagents (soluble carbohydrate, chimeric receptor, neutralizing antibody), could have engaged with and activated the phagocytes in unexpected ways that altered their phagocytic functions. In any of these cases, a lack of specificity and/or biological context led to the conclusion that Dectin-1 was likely important for *Cryptococcus*-spore interactions. Subsequent experiments using an array of more integrated, biologically relevant assessment tools conflict with that initial conclusion and now represent a more accurate picture of spore interactions with phagocytes.

This more accurate representation of spore-phagocyte interactions does not rule out the possibilities that the receptors tested are able to recognize *C*. *neoformans* spores and/or yeast but do so only in the presence of other co-receptors, in specific in vivo contexts, or in a concerted manner that masks the contributions of any one of them. Whether or not the specific receptors tested here are involved, there exist two primary models for the phagocytosis of *C*. *neoformans* spores. The first is that a large number of different receptors (including those tested here) may be responsible for spore phagocytosis, with no individual receptor primarily responsible (Fig 6B, left), similar to what is seen in the phagocytosis of *Mycobacterium bovis*, which is taken up through the engagement of TLR2, TLR4, SR-A and CD36 [36]. A second possibility is that a different, as yet untested, receptor may be the major player responsible for the identification and/or uptake of cryptococcal spores (Fig 6B, right). Possible receptors for spores include: toll-like receptors; scavenger receptors such as SR-A (which has been shown to interact with glucans) [37], and SCARF1 and CD36, which have been found previously to interact with *C*. *neoformans* yeast [38]. It is also possible that a currently unidentified spore-binding receptor may be responsible and emerge as new mammalian receptors are discovered.

As more mechanisms of receptor-ligand interactions are investigated, it will be critical to give extra consideration to inconsistencies observed between results generated using different methods. Complex, contextual challenges accompany these methods in part because immune cells are primed to react to exogenous materials, including the reagents used for experimentation. Phagocytes have been shown to react to foreign DNA and RNA (used for silencing or expression vectors) [39] and soluble carbohydrate ligands [40,41]. These immune responses could alter phagocytosis through mechanisms outside of the interaction that the experiment intends to block. Blocking carbohydrates have in fact shown different phenotypes with varied activation of phagocytes [42], and immune cells react inconsistently to different ligands targeting the same receptor [43]. Finally, compensatory mechanisms employed by phagocytes can mask phenotypic differences even when a receptor involved in phagocytosis is targeted, making it difficult to conclusively rule out the contributions of any given receptor [44].

The studies presented here conclusively show that Dectin-1, a receptor previously identified as likely mediating the majority *C*. *neoformans* spore phagocytosis, may play a role in spore binding, but is in fact not required for robust phagocytosis by host immune cells. This work emphasizes and exemplifies the importance of biological context at every scale when studying receptor-ligand interactions: from receptor presentation (membrane bound or soluble), the effects of exogenous reagents, to the other proteins and receptors represented in the system. Straying too far from the biological context in any of these areas may result in false positive or negative results. Phagocyte identity is an example of another important factor in biological context. The experiments in this study were carried out using phagocytes of disparate origin and immunological roles: alveolar macrophages, which are resident phagocytes of the lung and act as first responders, and dendritic cell and macrophage-enriched bone-marrow derived phagocytes, which serve as models of recruited inflammatory mononuclear cells that function in infection sites as distal effector cells [45]. The absence of a large phenotype in either phagocyte population gives weight to our conclusion that Dectin-1 is not essential for robust *Cryptococcus* spore uptake. However, this does not exclude the formal possibility that other phagocyte population subsets could exhibit alternative phenotypes for spore-receptor interactions, and questions investigating alternative/more specific biological contexts would necessitate experiments using more complex phagocyte derivation and identification protocols. While phagocytosis is a promising target for the development of antifungal immunomodulatory therapeutics, our work illustrates that identification of phagocytic receptors will require the integration of multiple approaches that carefully mimic biologically relevant contexts.

## Supporting information

S1 Fig Harvested alveolar macrophages display SiglecF+CD11c+CD64+Ly6G- phenotype. Alveolar macrophages were harvested by bronchoalveolar lavage of WT mice. Macrophages were stained with 1:150 dilutions of anti-mouse CD64 FITC, anti-mouse SiglecF PeCy7, anti-mouse Ly6G BUV395, anti-mouse CD11c BV786, Fc block and Live/Dead near IR. 89.0% of the live cells stained with the expected phenotype of alveolar macrophages as SiglecF+CD11c+CD64+Ly6G-.
(TIF)

S2 Fig Dectin-1 antibody can recognize HA-tagged Dectin-1 expressed on CHO cells. CHO-K1 cells were engineered to express Dectin-1 (Clec-7a) with a C-terminal HA tag. Dectin-1-HA protein localization and recognition by an antibody directed against Dectin-1 and conjugated to FITC (green) antibody was assessed. Cells were evaluated using both light and fluorescence microscopy at 1000X magnification. White bars represent 10 μm.
(TIF)

S3 Fig Dectin-1 is not sufficient to bind *C*. *neoformans* yeast. CHO-K1 cells expressing Dectin-1-HA protein were treated with an antibody directed against HA and conjugated to Cy3 (red). Visual assays were used to assess binding of heat-killed *C*. *neoformans* yeast stained with calcofluor white (blue). Cells were evaluated using both light and fluorescence microscopy at 1000X magnification. White bars represent 10 μm.
(TIF)

S4 Fig Schematic for BWZ/B3Z reporter cells. BWZ cells and a subline expressing Dectin-1-CD3ζ (Dectin-1), as well as B3Z cells expressing Dectin-2, Mincle, FcRγ chain, Dectin-2 + FcRγ, Mincle + FcRγ, or MCL+ FcRγ can be stimulated with microbes. If a receptor is engaged and activated on a reporter cell, downstream signaling will lead to LacZ expression. After 18 hours, ß-galactosidase activity is measured using a colorimetric assay and expressed as relative OD 560/620 values.
(TIF)

S5 Fig Dectin-1, Dectin-2, Mincle and MCL do not recognize *C*. *neoformans* yeast. BWZ and B3Z cells and sublines expressing FcRγ chain only (FcR), Dectin-2 and FcRγ chain together (Dec-2+FcR), Mincle and FcRγ chain together (Mincle+FcR), MCL and FcRγ together (MCL+FcR) and Dectin-1-CD3ζ (Dec-1) were stimulated an with 30 heat-killed *C*. *neoformans* yeast per reporter cell. After 18 hours of co-incubation, ß-galactosidase activity was measured using a colorimetric assay and expressed in absorbance units (AU) on the y-axis. Data shown are the mean ± the standard deviation of duplicate wells of a single experiment and are representative of three independent experiments.
(TIF)

S6 Fig Calcofluor white staining following phagocytosis of GFP-expressing *C*. *neoformans* spores can be used to differentiate phagocytosis from binding. DC enriched-bone marrow-derived phagocytes were allowed to phagocytose GFP-expressing *C*. *neoformans* spores. Subsequent staining with calcofluor white identified fungal cells remaining extracellular. For WT, Dectin-1, and Dectin-2 phagocytes, this staining protocol allowed for the differentiation of phagocytosed (GFP+CFW-) and extracellularly bound (GFP+CFW+) *C*. *neoformans* spores.
(TIF)

S7 Fig DC-enriched bone marrow-derived phagocytes and AMs remain GFP and calcofluor white negative in the absence of fungal cells or when incubated with phagocytic resistant *C*. *neoformans* yeast. Flow cytometry results looking at the intensity of GFP and Calcofluor white in populations of DC-enriched phagocytes **(A)** and AMs (**B)** in the absence of cryptococcal cells (left) or incubated with live *C*. *neoformans* yeast (right), which are known to resist phagocytosis. As anticipated, the phagocyte cell populations observed were overwhelmingly GFP and calcofluor white negative.
(TIF)

S8 Fig Dectin-1 and Dectin-2 knockout AMs show decreased abilities to bind spores compared to WT AMs; both Dectin-1 and Dectin-2 knockouts show increased phagocytosis of bound spores by AMs and DC-enriched bone marrow-derived phagocytes compared to WT cells. Analysis of flow cytometry results to separately assess binding (GFP+ phagocytes/total phagocytes) and phagocytosis (GFP+CFW- phagocytes / GFP+ phagocytes) of spores by bone marrow-derived phagocytes **(A, B)** and AMs **(C, D)**. Spores bound to knockout phaocytes with the same frequency as WT cells. Bound spores were phagocytosed by Dectin-1 and Dectin-2 knockout phagocytes more frequently than WT cells. Dectin-1 and Dectin-2 knockout AMs showed a decreased ability to bind spores. Spores bound to knockout AMs were phagocytosed more frequently than those bound to WT cells. Bar graphs show average values, and error bars represent the standard error of the mean.
(TIF)

S1 Table Summary of experiments using heat-killed or live *C*. *neoformans* cells. (DOCX)
