# Supplementary figures and images for: Characterization of C-type lectins reveals an unexpectedly limited interaction between Cryptococcus neoformans spores and Dectin-1
Source: PLoS One. 2017 Mar 10;12(3):e0173866. doi: 10.1371/journal.pone.0173866 (PMC5345868; doi:10.1371/journal.pone.0173866)

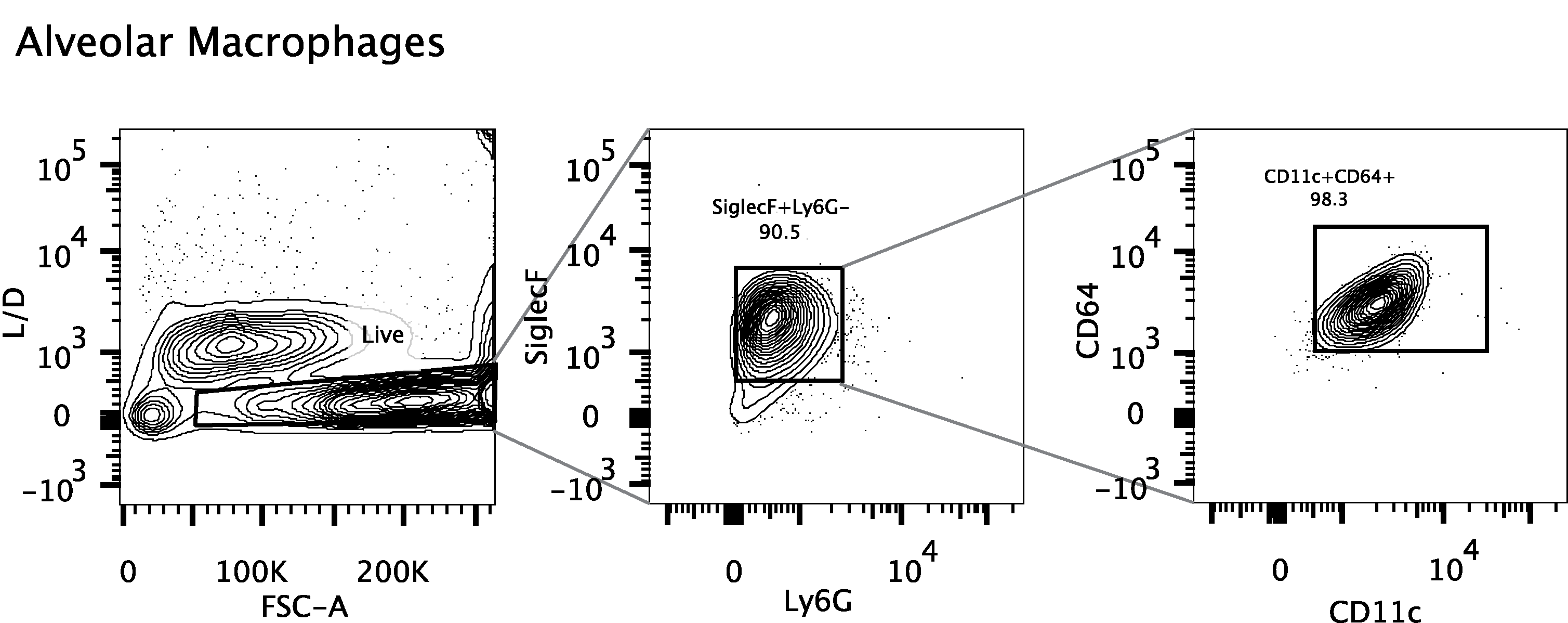

Supplement: S1 Fig — Alveolar macrophages were harvested by bronchoalveolar lavage of WT mice. Macrophages were stained with 1:150 dilutions of anti-mouse CD64 FITC, anti-mouse SiglecF PeCy7, anti-mouse Ly6G BUV395, anti-mouse CD11c BV786, Fc block and Live/Dead near IR. 89.0% of the live cells stained with the expected phenotype of alveolar macrophages as SiglecF+CD11c+CD64+Ly6G-. (TIF) [file pone.0173866.s001.tif]

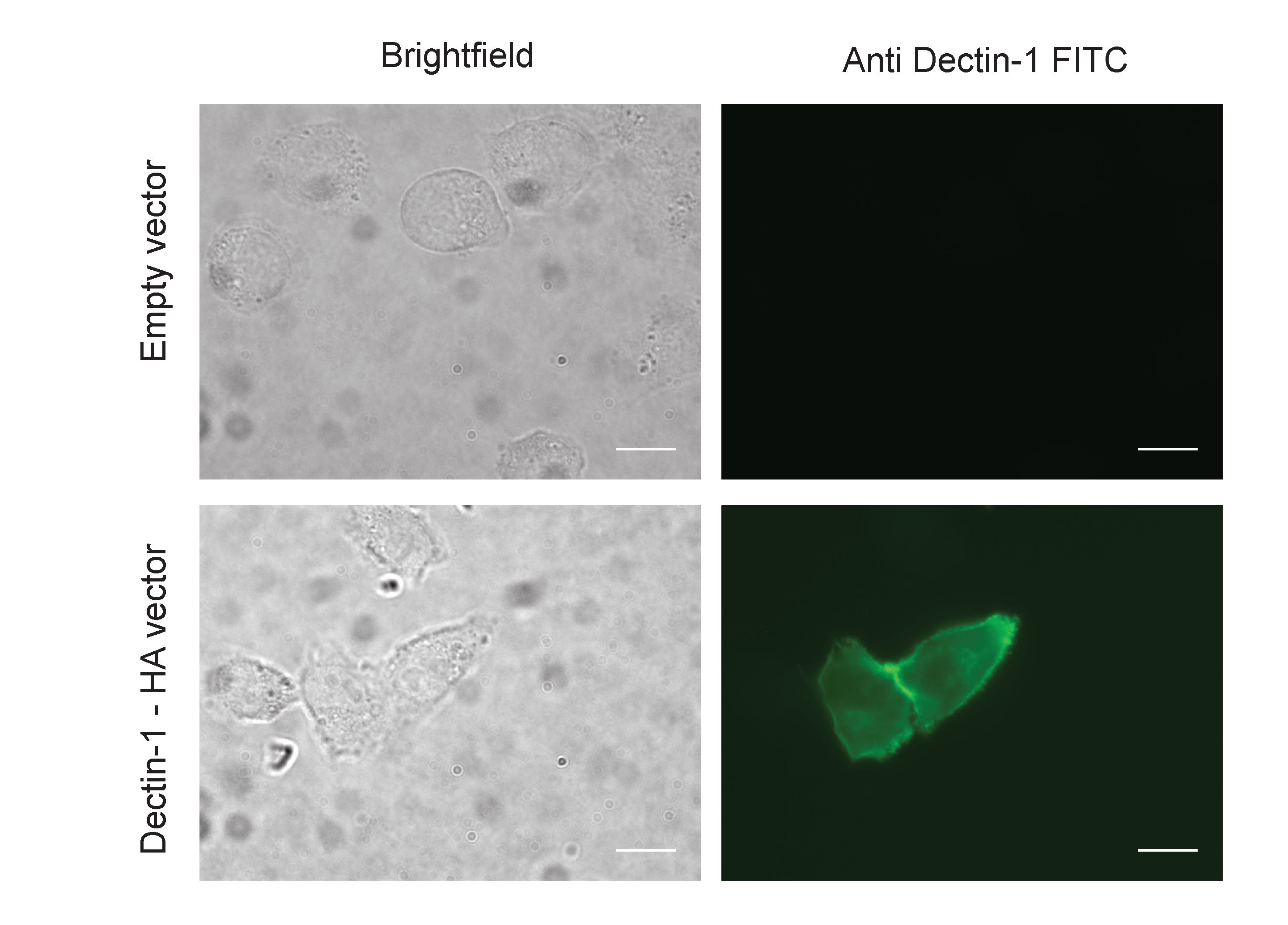

Supplement: S2 Fig — CHO-K1 cells were engineered to express Dectin-1 (Clec-7a) with a C-terminal HA tag. Dectin-1-HA protein localization and recognition by an antibody directed against Dectin-1 and conjugated to FITC (green) antibody was assessed. Cells were evaluated using both light and fluorescence microscopy at 1000X magnification. White bars represent 10 μm. (TIF) [file pone.0173866.s002.tif]

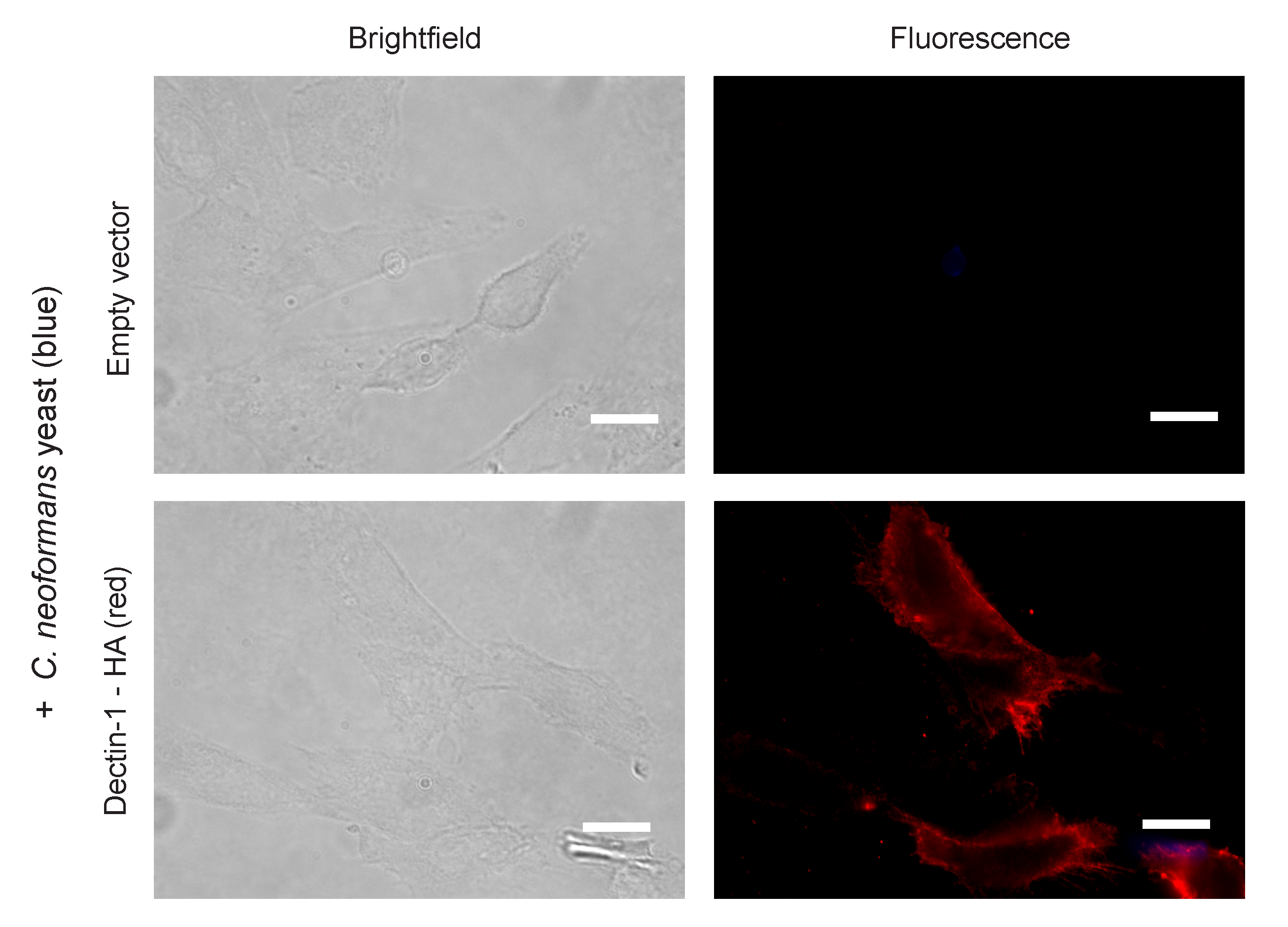

Supplement: S3 Fig — CHO-K1 cells expressing Dectin-1-HA protein were treated with an antibody directed against HA and conjugated to Cy3 (red). Visual assays were used to assess binding of heat-killed C. neoformans yeast stained with calcofluor white (blue). Cells were evaluated using both light and fluorescence microscopy at 1000X magnification. White bars represent 10 μm. (TIF) [file pone.0173866.s003.tif]

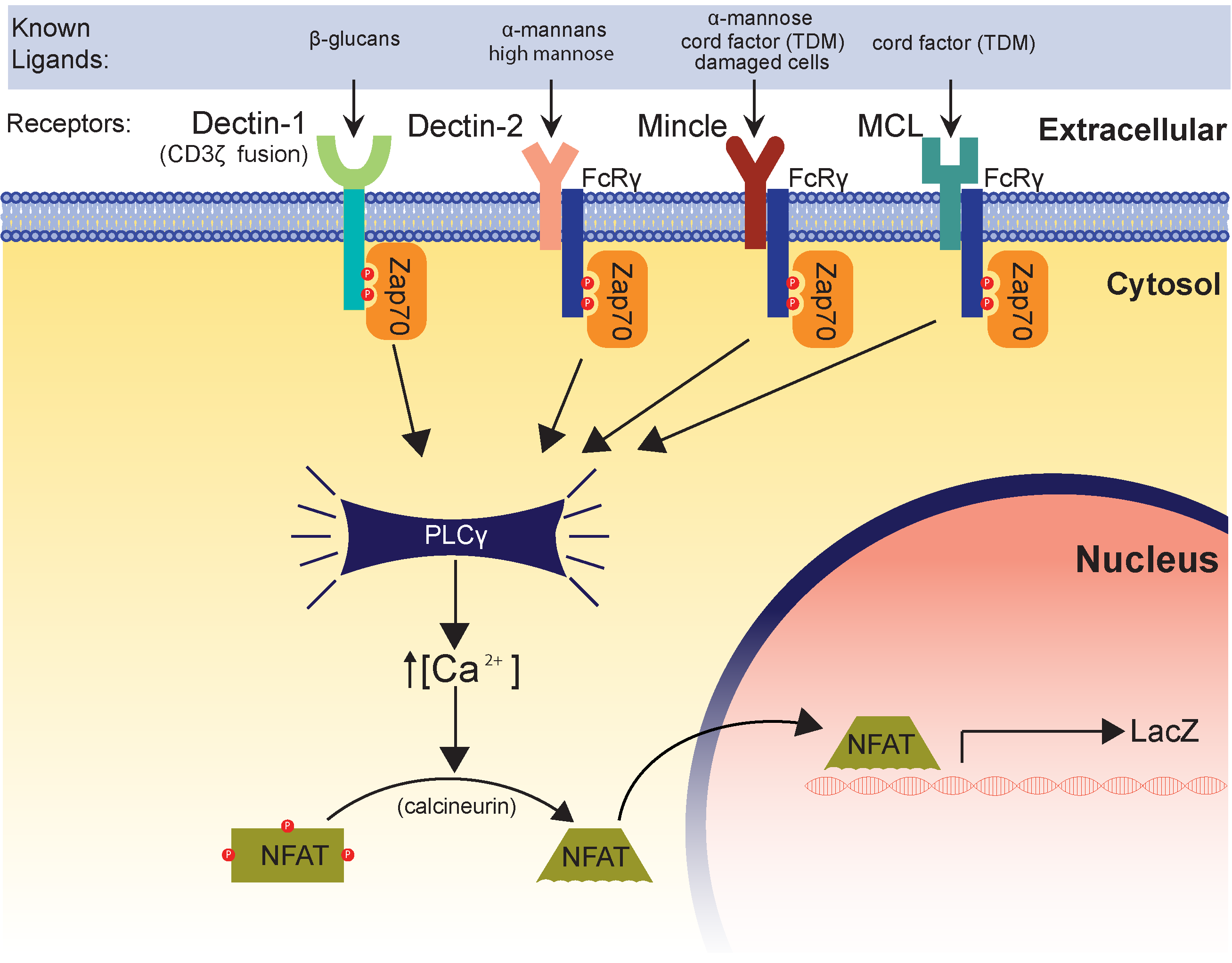

Supplement: S4 Fig — BWZ cells and a subline expressing Dectin-1-CD3ζ (Dectin-1), as well as B3Z cells expressing Dectin-2, Mincle, FcRγ chain, Dectin-2 + FcRγ, Mincle + FcRγ, or MCL+ FcRγ can be stimulated with microbes. If a receptor is engaged and activated on a reporter cell, downstream signaling will lead to LacZ expression. After 18 hours, ß-galactosidase activity is measured using a colorimetric assay and expressed as relative OD 560/620 values. (TIF) [file pone.0173866.s004.tif]

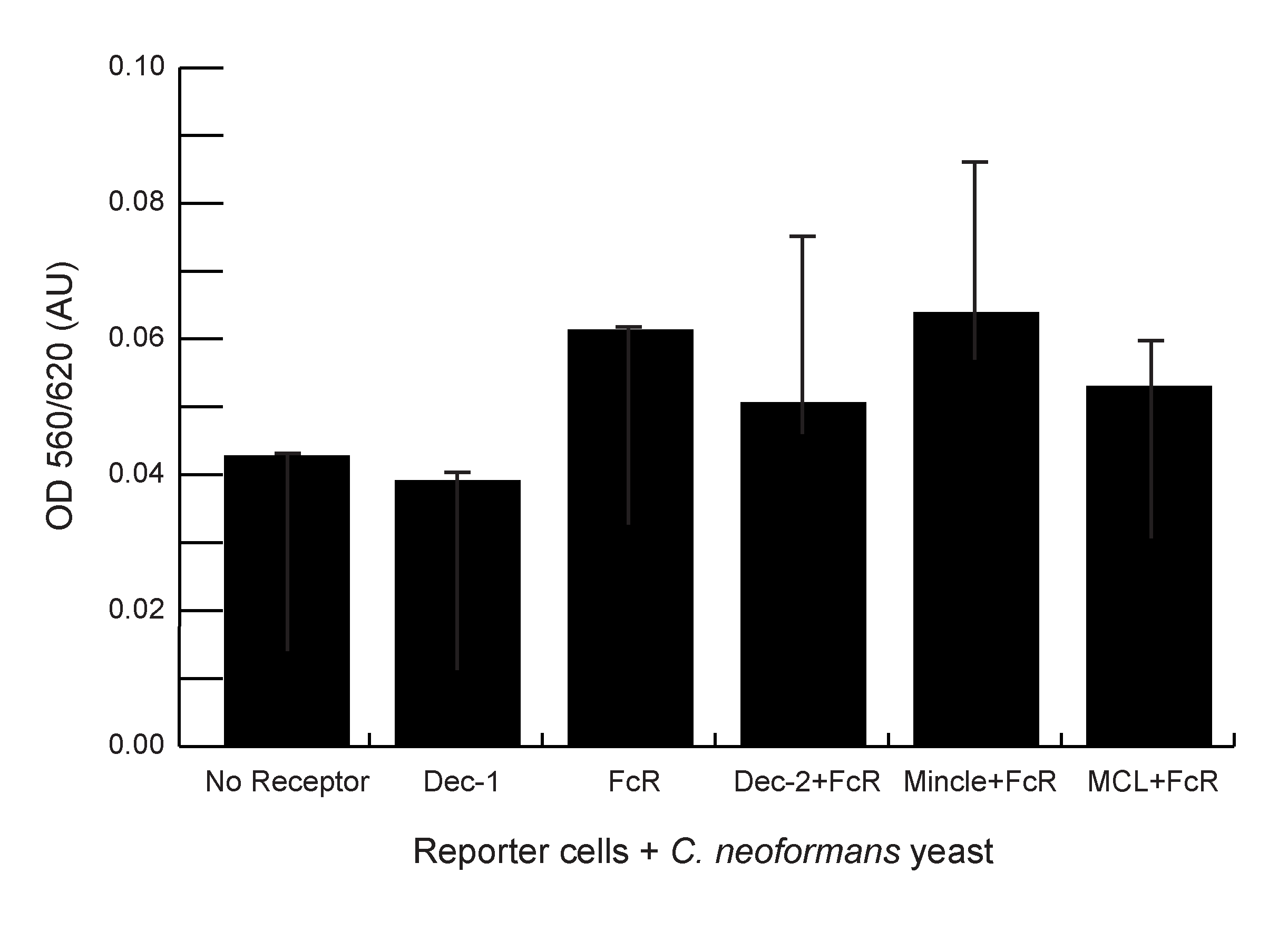

Supplement: S5 Fig — BWZ and B3Z cells and sublines expressing FcRγ chain only (FcR), Dectin-2 and FcRγ chain together (Dec-2+FcR), Mincle and FcRγ chain together (Mincle+FcR), MCL and FcRγ together (MCL+FcR) and Dectin-1-CD3ζ (Dec-1) were stimulated an with 30 heat-killed C. neoformans yeast per reporter cell. After 18 hours of co-incubation, ß-galactosidase activity was measured using a colorimetric assay and expressed in absorbance units (AU) on the y-axis. Data shown are the mean ± the standard deviation of duplicate wells of a single experiment and are representative of three independent experiments. (TIF) [file pone.0173866.s005.tif]

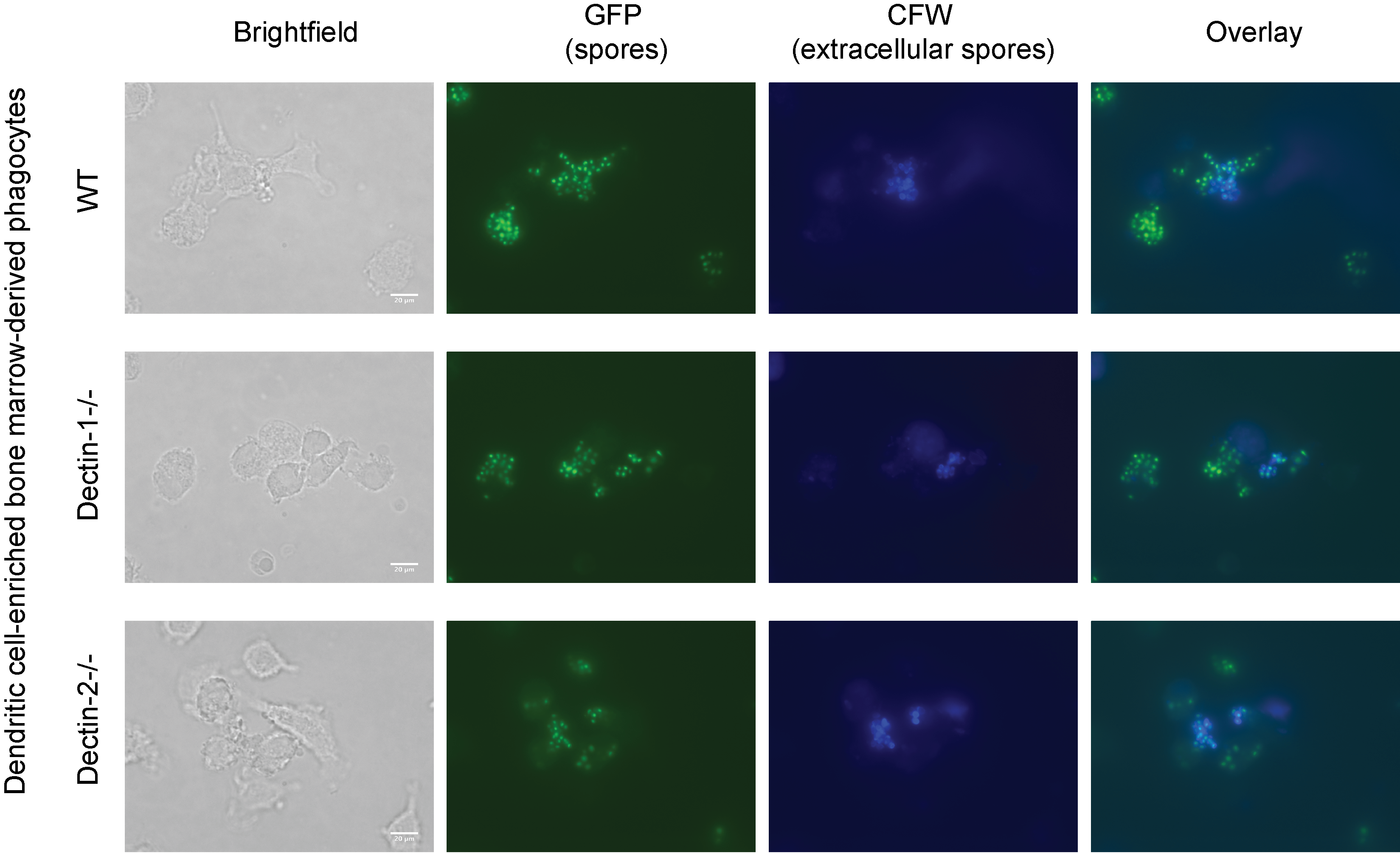

Supplement: S6 Fig — DC enriched-bone marrow-derived phagocytes were allowed to phagocytose GFP-expressing C. neoformans spores. Subsequent staining with calcofluor white identified fungal cells remaining extracellular. For WT, Dectin-1, and Dectin-2 phagocytes, this staining protocol allowed for the differentiation of phagocytosed (GFP+CFW-) and extracellularly bound (GFP+CFW+) C. neoformans spores. (TIF) [file pone.0173866.s006.tif]

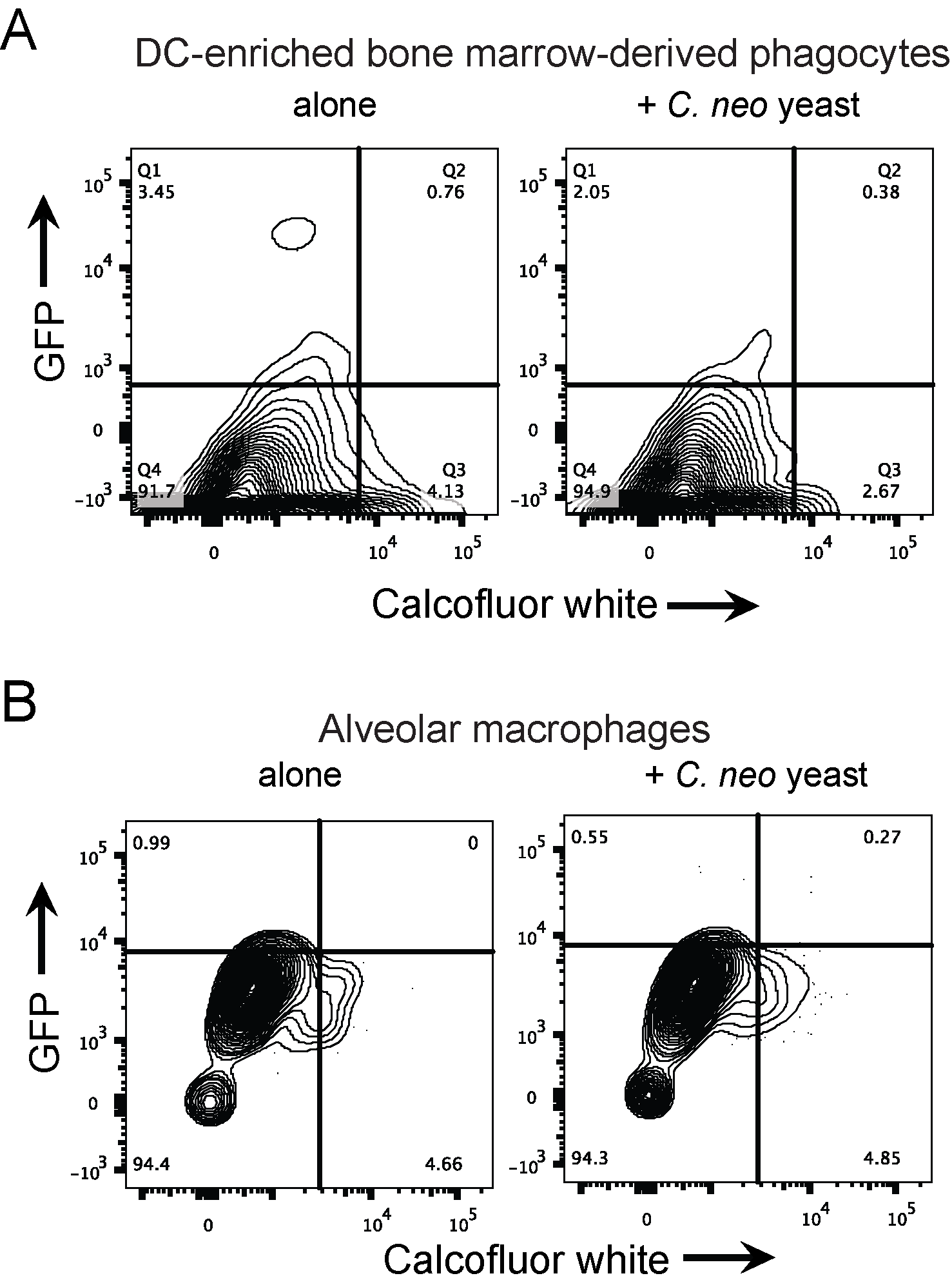

Supplement: S7 Fig — Flow cytometry results looking at the intensity of GFP and Calcofluor white in populations of DC-enriched phagocytes (A) and AMs (B) in the absence of cryptococcal cells (left) or incubated with live C. neoformans yeast (right), which are known to resist phagocytosis. As anticipated, the phagocyte cell populations observed were overwhelmingly GFP and calcofluor white negative. (TIF) [file pone.0173866.s007.tif]

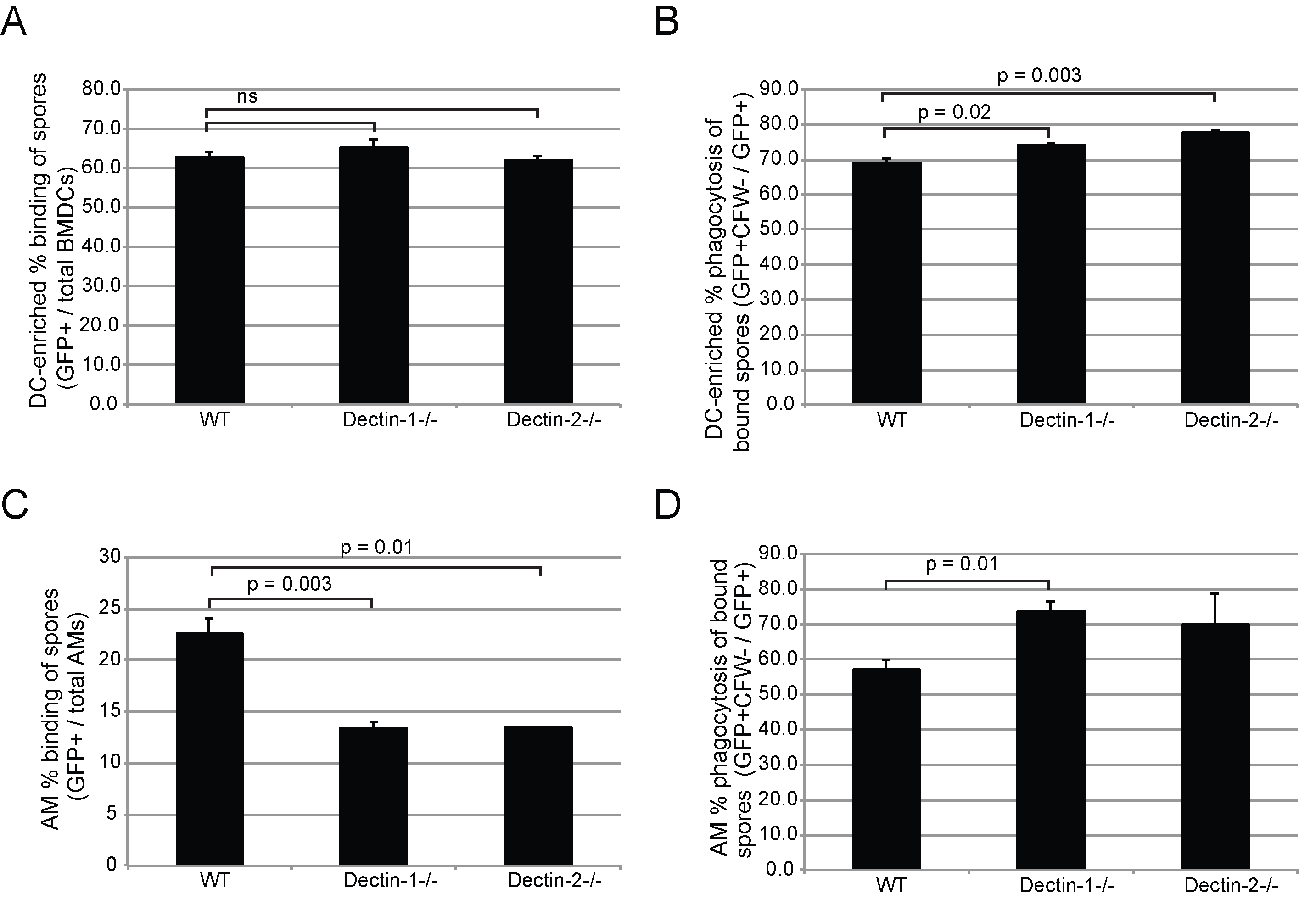

Supplement: S8 Fig — Analysis of flow cytometry results to separately assess binding (GFP+ phagocytes/total phagocytes) and phagocytosis (GFP+CFW- phagocytes / GFP+ phagocytes) of spores by bone marrow-derived phagocytes (A, B) and AMs (C, D). Spores bound to knockout phaocytes with the same frequency as WT cells. Bound spores were phagocytosed by Dectin-1 and Dectin-2 knockout phagocytes more frequently than WT cells. Dectin-1 and Dectin-2 knockout AMs showed a decreased ability to bind spores. Spores bound to knockout AMs were phagocytosed more frequently than those bound to WT cells. Bar graphs show average values, and error bars represent the standard error of the mean. (TIF) [file pone.0173866.s008.tif]
