# Supplementary material for: Characterization of C-type lectins reveals an unexpectedly limited interaction between Cryptococcus neoformans spores and Dectin-1
Source: PLoS One. 2017 Mar 10;12(3):e0173866. doi: 10.1371/journal.pone.0173866 (PMC5345868; doi:10.1371/journal.pone.0173866)
Supplement: S1 Table — (DOCX) [file pone.0173866.s009.docx]

**S1 Table. Summary of experiments using heat-killed or live *C. neoformans* cells.**

| **Figure/Experiment** | **Heat-killed** | **Live** |
| --- | --- | --- |
| Fig. 1: CHO Cell binding assay | X  (qualitative, Fig. 1A and 1B) | X  (quantitative, Fig 1C) |
| Fig. 2: Heterologous reporter cell assay | X |  |
| Fig. 3:Assocaition with bone-marrow derived macrophages |  | X |
| Fig. 4: Binding/phagocytosis with BMDCs and AMs |  | X |
| Fig. 5: Mouse survival |  | X |
| S5 Fig: Microscopy of binding vs. phagocytosis staining |  | X |
